# Development and nutritional evaluation of an extruded grain amaranth-instant porridge as a supplementary food for children under five with moderate acute malnutrition

**DOI:** 10.3389/fnut.2026.1895788

**Published:** 2026-09-14

**Authors:** Amos Steven Mwikwabe, Haikael David Martin, Benson Kusirie Mushi, Dyness Dickson Kejo

**Affiliations:** 1 School of Life Sciences and Bio Engineering, Nelson Mandela African Institution of Science and Technology (NM-AIST), Arusha, Tanzania; 2 Department of Post-Harvest Technology, Tanzania Agricultural Research Institute (TARI), Tengeru-Arusha, Tanzania; 3 Tanzania Food and Nutrition Centre (TFNC), Dar es Salaam, Tanzania; 4 Department of Food Science and Technology, University of Dar es Salaam, Dar es Salaam, Tanzania

**Keywords:** child nutrition, extrusion cooking, grain amaranth, instant porridge, moderate acute malnutrition, nutrient density, ready to use supplementary food, supplementary foods

## Abstract

Moderate acute malnutrition (MAM) among children under five remains a challenge in Tanzania, where the fortified blended foods used to manage it are almost entirely imported, expensive and inaccessible. This study developed and evaluated a grain amaranth-based extruded instant porridge as a locally sourced alternative. Five formulations were generated from grain amaranth, pro-vitamin A maize, soybean and sesame by two-stage linear programming using regional food composition data, with crude protein maximized subject to a technological fat ceiling of 11.0 g/100 g. Formulation 1, the least-cost solution, and Formulation 5, the most nutrient-dense within fat ceiling that is more economical than the non-compliant Formulation 3, were advanced to twin-screw extrusion. Proximate composition (AOAC 2005), mineral content (microwave plasma atomic emission spectrometry) and anti-nutritional factors (colorimetric methods) were determined; functional and sensory properties were evaluated; data were analyzed by ANOVA. Protein ranged from 18.14 g/100 g in Formulation 1 to 20.53 g/100 g in Formulation 5 reaching 98.0% of the WFP/UNICEF Super Cereal Plus (CSB++) minimum of 400 kcal/100 g. Formulation 5 showed the highest fat (11.95 g/100 g) and zinc (8.98 mg/100 g), all within CSB++ ranges, with only potassium below specification (37.1%). Extrusion significantly increased water absorption capacity (*p* < 0.001), shifted the flour from the fair to good flowing category, and reduced tannins, oxalates and phytate by 50.2, 64.9 and 77.6% relative to raw grain amaranth. Sensory scores showed good overall acceptability (6.2–6.9), with significant differences for aroma (*p* < 0.001) and flavor (*p* = 0.002); SC(F5)-777 ranked highest among extruded samples. Food-to-food fortification using grain amaranth at 40–45% produced energy- and nutrient-dense instant porridge with improved mineral content, functional properties, and sensory characteristics, demonstrating strong potential for use as locally available supplementary food for managing MAM in children under five.

## 1 Introduction

Malnutrition remains one of the most persistent global public health challenges, particularly in low- and middle-income countries. Although it affects individuals across all age groups, the most vulnerable populations are children under 5 years, pregnant and lactating women, and the elderly (1). Malnutrition manifests in two primary forms: under-nutrition characterized by wasting, stunting, underweight, and micro nutrient deficiencies and over-nutrition, including overweight and obesity (2). Children suffering from acute under-nutrition face an elevated risk of morbidity, mortality, and recurrent or prolonged illness further exacerbates wasting, stunting, and micro-nutrient deficiencies. Globally, an estimated 149 million children under five are stunted, 45 million are wasted, and 37 million are overweight or obese (3, 4).

In Tanzania, under-nutrition remains a significant public health concern despite ongoing interventions. According to the Tanzania Demographic and Health Survey 2022, 30% of children under five are stunted, 3% are wasted, and over 50% suffer from iron deficiency anemia (5). Moderate Acute Malnutrition (MAM) is particularly problematic because children with MAM have increased nutritional needs relative to their healthy peers and are at heightened risk of progressing to Severe Acute Malnutrition (SAM) if not managed appropriately (6). Treatment of SAM is resource-intensive, often requiring hospitalization and specialized therapeutic foods, with recovery costs reaching as high as more than US$200 per child and an average hospital stay of 27 days (7). These financial and logistical burdens contribute to treatment dropouts and increase preventable mortality.

Despite efforts such as the Shirati Peanut Project and Orange-Fleshed Sweet Potato programs (8), under-nutrition persists due to widespread poverty, food insecurity, and limited access to nutrient-dense complementary foods (9). Many households are unable to provide adequate energy, protein, and micro-nutrients for their children, resulting in diets insufficient to meet growth and immunity demands (10). Current therapeutic options are insufficiently accessible, largely imported, and often expensive. Peanut-based RUTFs, while effective, present additional issues: high susceptibility to aflatoxin contamination, short shelf life due to their semi-solid nature, and limited affordability or availability in rural areas, it is with this regard that leads to the exploration of grain amaranth in the combating malnutrition among children under 5 years of age (11).

Addressing MAM requires innovative, context-appropriate solutions that strengthen dietary adequacy using locally available, affordable, and nutrient-dense foods. Grain amaranth (*Amaranthus* spp.) is an indigenous crop with considerable potential in this regard. It contains high-quality protein rich in essential amino acids such as lysine and methionine nutrients generally lacking in staple cereals and provides substantial quantities of calcium, iron, zinc, and dietary fiber (11). Despite these advantages, grain amaranth remains underutilized due to limited awareness and inadequate processing technologies. Emerging evidence indicates that amaranth-based products can improve nutritional outcomes in malnourished children (12), yet there is a scarcity of locally produced instant foods suitable for integration into MAM management programs. Given the high cost and dependence of imported ingredients associated with commercial RUTFs, treatment of SAM represents an economic burden for an average family and health systems in low income countries (7), escalate the growing interest in developing an affordable amaranth-based product presents a viable nutrition-sensitive intervention.

The objective of this study was to develop and characterize an energy and nutrient- dense instant porridge (ENDIP) from grain amaranth and complementary locally available crops, and to evaluate it against the WFP/UNICEF Super Cereal Plus specification. Specifically, the study aimed to (i) derive candidate formulations by Linear programming under simultaneously nutritional, technological and cost constraints; (ii) to determine the proximate and mineral composition of extruded products and their compliance with CSB++ specification; (iii) quantify the effect of blending and extrusion on tannin, oxalate and phytate content and the resulting phytate-to-mineral molar ratios; (iv) characterize the functional properties governing reconstitution and handling; and (v) assess sensory acceptability among caregivers. To meet these objectives, a nutrient-dense with improved nutrients bio-availability and functional properties compared to conventional cereal-based porridge while maintaining acceptable sensory properties for community use was developed.

## 2 Materials and methods

### 2.1 Materials

The Akeri variety of grain amaranth (*Amaranthus cruentus*) was obtained from the Tanzania Agricultural Research Institute (TARI). This variety was bred by Dr. Fekadu F. Dinssa of the World Vegetable Center (World-Veg), Arusha, Tanzania, and officially released in Tanzania by TARI under registration certificate number TOSCI/180/2017 (13). While pro-vitamin A maize, sesame and soybean were sourced from local markets in Arusha Tanzania, Selection criteria for all ingredients included nutrient density, local availability, affordability, and cultural acceptability.

### 2.2 Preparation

All grains and ingredients were first sorted and cleaned to remove stones, debris, and dust. Grain amaranth and maize were sieved using a grain cleaning machine equipped with a 0.7 mm upper sieve and a 0.5 mm lower sieve. Maize and other ingredients were subsequently washed to remove residual sand and dust. Following cleaning, the maize and amaranth grains were sun-dried to approximately 12% moisture content, while other ingredients were dried to a moisture level suitable for milling. Soybeans were boiled for 45 min, mechanically de-hulled, and sun-dried over 2 days (approximately 7 h per day) to reduce anti-nutritional factors. All prepared ingredients were then weighed and blended according to the designed ratios to form a composite mixture ready for grinding and subsequent processing into the formulations as shown in Figure 1.

**Figure 1:**
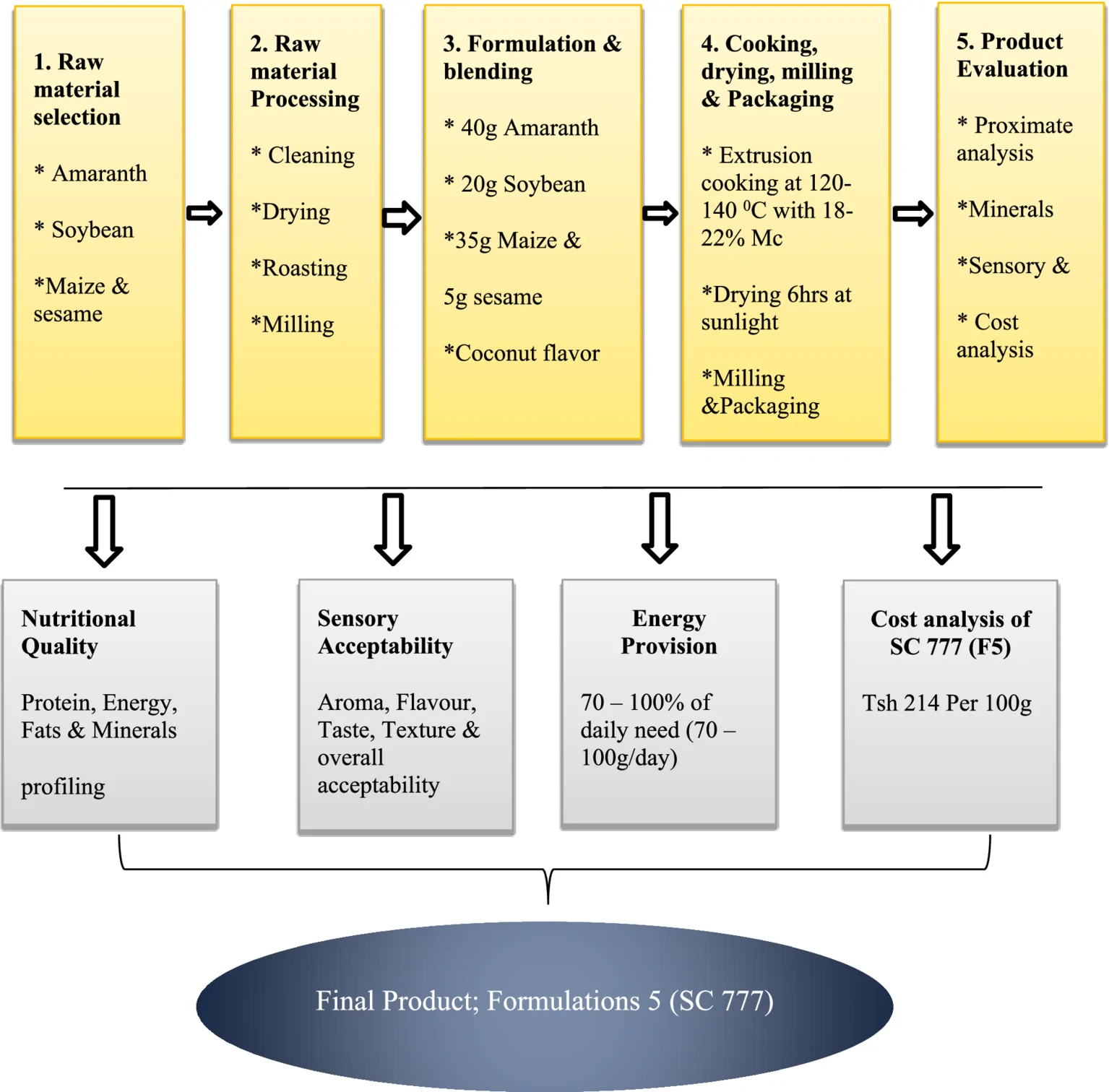
Product development flow for coconut flavored amaranth instant porridge (Formulation 5- SC 777).

### 2.3 Product formulation, milling and extrusion

The theoretical formulation of the nutrient-dense instant porridge flour (ENDIP) was done using nutrient information from the Tanzanian and Kenyan Food Composition Table (14, 15), with the goal of meeting the dietary requirements of children with MAM. The product was designed to be delivered as a 100 g/day ration, which provides a meaningful energy and micronutrient supplement while remaining within the volume a child with MAM can realistically consume alongside the home diet (16, 17). Linear programming was employed to optimize nutrient combinations; optimization was done using Microsoft Excel in two stages. The decision variables were the mass fraction of grain amaranth (x1), pro- vitamin A maize (x2), soybean (x3) and sesame (x4), subject to x1 + x2 + x3 + x4 = 100. In the nutrient stage the objective function was to maximize crude protein to contribute at least 11% of the total kcal/100 (max P1 = Σ (pᵢ xᵢ), subject to technological constraint on fat, Fat = Σ (fᵢ xᵢ)/100 ≤ 11.0 g/100 g), where fᵢ is the fat content (g/100 g) of ingredient i and xᵢ its mass fraction. The procedure generated five formulations (Table 1), in which grain amaranth ranged from 31 to 41%, pro-vitamin A maize from 35 to 45%, soy bean from 15 to 21% and sesame from 5 to 11%. Energy content ranged from 369 to 384.9 kcal/100 g, protein from 14.6 to 16.6 g/100 g (equivalent to 15–17.3% of total energy), fat from 9.6 to 12.8 g/100 g, ash from 2.7–3.1 g/100 g and fiber from 10.3–11.1 g/100 g.

**Table 1:** Theoretical nutrient composition and ingredient ratios of the five formulations calculated from the Tanzania Food Composition Tables (14, 15).

Description	F1	F2	F3	F4	F5
Ingredient ratio (%)					
Grain amaranth	35	41	31	41	40
Pro-vitamin A maize	45	39	37	38	35
Soybean	15	15	21	16	20
Sesame	5	5	11	5	5
Nutrients composition (/ 100 g)					
Energy (kcal)	369.3	370	384.9	370	372.1
Protein (g)	14.6	14.9	16.6	15.3	16.2
Fat (g)	9.6	9.6	12.8	9.7	10.3
Ash (g)	2.7	2.8	3.1	2.8	3.0
Fiber (g)	10.5	10.4	11.1	10.3	10.9
Selection criteria					
Cost (TZS/100 g)	192.8	205.4	215.9	207.7	214.8
Nutritional constraints met	✓	✓	✓	✓	✓
Technological constraints met	✓	✓	✗	✓	✓
Selected for extrusion	✓	✗	✗	✗	✓

Formulation cost was computed as Cost = Σ [(% inclusion / 100) × (ingredient price/100 g)], using April 2026 prevailing Arusha domestic market prices of 700, 3,000, 3,650, and 2,800 TZS/kg for maize, soybean, sesame and grain amaranth, respectively, and ranged from 192.8 TZS/100 g (F1) to 215.9 TZS/100 g (F3).

Formulation 3 was the only formulation to exceed the fat ceiling (12.8 g/100 g) but also most expensive formulation and was therefore not advanced to extrusion, together with F2 and F4, which had lower protein (14.9 and 15.3 g/100 g respectively) than the remaining formulations. Finished-product cost was obtained by adding to the raw-material cost an extrusion processing fee of 1,050 TZS/kg, being the rate charged for the pilot extrusion runs conducted in this study, and packaging at 250 TZS per one-kilogram pack.

Formulations 1 and 5 were selected for extrusion. Ingredients for each formulation were weighed according to the designed ratios and milled using a hammer mill fitted with a 0.5-mm sieve to produce uniform composite flour. The flours were then processed using extrusion technology at Sokoine University of Agriculture (SUA). During extrusion, the composite flour was mixed with 0.98–1.5 kg of water per 10 kg of flour to form 18–22% moisture contents dough, which was fed into a double-screw extruder at a processing speed of 30 rpm and feeder rate of 20-21 kg/h. Extrusion was carried out at temperatures ranging from 120 °C to 140 °C to produce nutrient-rich that could be reconstituted with hot water into an instant porridge suitable for children under five with MAM.

### 2.4 Functional properties: water absorption capacity and bulk density

#### 2.4.1 Water absorption capacity (WAC) test

The water absorption capacity (WAC) of un-extruded and extruded grain amaranth formulations (ENDIP) was determined following the method of Yamazaki (1953) (18), with minor modifications to accommodate the hydration properties of amaranth flour. Briefly, 20 g of each formulation was weighed into a container, and 60 mL of distilled water was added. The mixture was stirred manually for 1 min to ensure uniform dispersion and complete wetting, then allowed to stand at 25 °C for 30 min to enable full hydration and swelling of the starch-protein matrix, the unbound water was gently decanted, leaving only the hydrated flour. After the incubation period, the total weight of the hydrated sample (flour plus absorbed water) was recorded. WAC was expressed as grams of water absorbed per gram of dry sample, calculated using the formula:


$$\mathrm{WAC}=\frac{W_{\text{hydrated sample}}-W_{\mathrm{dry}\;\text{sample}}}{W_{\mathrm{dry}\;\text{sample}}}$$

Where; *W_dry sample_*​ is the initial weight of the flour, and *W_hydrated sample_* is the weight of the sample after absorbing water. All measurements were performed in triplicate for both Formulation 1 and Formulation 5 in their un-extruded and extruded states.

#### 2.4.2 Determination of bulk density and flow indices

Loose bulk density, tapped density and derived flow indices were determined in triplicate for both cooked and uncooked flour samples, following procedures described by Onwuka (2005) (19), a 500 mls graduated cylinder (458.81 g(w1)) were used filled with flour and leveled gently with spatula to 100 mls (V) mark then weighed to record W2 (weight of a cylinder + flour). The cylinder was then tapped 50 times at a height of 5 cm until settled volume readings, then Volume attained at constant level were recorded (Vt) and finally flow indices were calculated as follows;


TD=(W2−W1)/Vt

BD=(W2−W1)/V

$$\mathrm{Car}r^{\prime }s\;\text{index}=[(\mathrm{TD}-\mathrm{BD})/\mathrm{TD}]\times 100$$

Hausner ratio=TD/BD

Where; BD is the bulk density, TD is the tapped density, W1 is the weight of an empty cylinder, W2 is the weight of a cylinder and flour, and V is the volume of flour before tapping while Vt is the volume of flour after tapping.

### 2.5 Nutrients analysis

Triplicate samples (25 g each) of the formulated product were collected and sealed in polythene bags for laboratory analysis. Nutrient composition was assessed to determine conformity with World Health Organization (WHO) recommendations for foods intended for the management of moderate acute malnutrition among children under 5 years. Both macro- and micronutrient parameters were quantified using standard analytical procedures, and the specific nutrients analyzed are described below.

#### 2.5.1 Proximate analysis

Standard Association of Official Analytical Chemist (AOAC, 2005) methods were used to determine: moisture content, ash content, crude fiber, crude protein (Kjeldahl method), crude fat (Soxhlet extraction) (20, 21), carbohydrates (by difference method) following the FAO-recommended procedure for food-composition work (22). Moisture content was determined by oven drying at 105 °C for 3 h following AOAC Official Method 925.10 (20). Ash content was measured by incinerating the sample at 550 °C in a muffle furnace for 5 h to constant weight, according to the procedure described by Thiex et al., (23). Crude fiber was analyzed following AOAC Official Method 978.10, which involves sequential acid and alkali digestion. Protein content was determined using the Kjeldahl method (AOAC Official Method 2001.11). Fat content was measured using the Soxhlet extraction technique according to AOAC Official Method 920.39 (21). Carbohydrate content was calculated by difference obtained from subtracting fats, protein, ash, fiber and moisture from 100.

#### 2.5.2 Mineral contents determination

Mineral contents (mg/100 g) including; potassium (K), sodium (Na), zinc (Zn), iron (Fe), magnesium (Mg) and calcium (Ca) were determined for the two selected formulations (Formulation 1 and Formulation 5) following AOAC (2005) procedures and the updated AOAC 984.27 (20) microwave digestion/ICP method as described by Poitevin et al., (24). Briefly, duplicate samples (2 g each) were digested with 65% nitric acid (HNO₃) for 1 h, followed by the addition of 500 μL hydrogen peroxide (H₂O₂). The digests were then diluted to 100 mL with deionized water. A 50 mL aliquot of each digest was analyzed using an Agilent MP-AES 4200 for mineral quantification.

### 2.6 Anti-nutrients content analysis

Anti-nutritional factors, namely tannins, oxalates, and phytates, were quantified in both raw and cooked samples to evaluate the effect of processing on the reduction of these compounds. Tannins were determined using the vanillin–HCl colorimetric method as described by Price et al. (25). Oxalate content was analyzed following AOAC Official Method 974.24, which involves acid extraction and titrimetric determination. Phytate content was determined according to AOAC Official Method 986.11 (20) using the Wade reagent colorimetric assay. All analyses were performed in duplicate to ensure analytical accuracy and reproducibility. Quantification of these anti-nutritional factors was essential for assessing the extent to which processing enhances mineral and nutrient bioavailability, thereby supporting the nutritional suitability of the formulated product for the management of moderate acute malnutrition in children under 5 years of age.

Phytate-to-mineral molar ratios were calculated for the raw blends and the extruded products to estimate the likely bioavailability of the minerals reported in Table 2. Phytate and mineral concentrations (mg/100 g) were converted to millimoles per 100 g by dividing each by its molar mass (phytic acid 660.04; calcium 40.08; iron 55.85; zinc 65.38 g/mol), and the phytate: iron, phytate: zinc and phytate: calcium ratios were obtained by division; being dimensionless, these are independent of the concentration units used. Ratios were interpreted against published critical values above which absorption of the mineral concerned is considered impaired, namely 1 for phytate: iron, 5 for phytate: zinc, 0.24 for phytate: calcium (26).

**Table 2:** Nutrients composition analysis for F1 and F5.

S. No	Nutrient per 100 g	Units	F1	F5
1	Total energy (kcal)	Kcal/100 g	379.67	391.93
2	Protein (g)	g/100 g	18.14	20.53
3	Fats	g/100 g	8.75	11.95
4	Calcium (Ca)	mg/100 g	637.94 ± 0.02	587.86 ± 0.02
5	Magnesium	mg/100 g	312.35 ± 0.39	174.96 ± 0.39
6	Iron (Fe)	mg/100 g	5.43 ± 0.003	11.44 ± 0.003
7	Potassium (K)	mg/100 g	98.71 ± 0.24	240.92 ± 0.24
8	Sodium (Na)	mg/100 g	297.62 ± 2.95	226.57 ± 2.95
9	Zinc (Zn)	mg/100 g	8.07 ± 0.026	8.98 ± 0.026

Where F1 stands for formulation 1 and F5 for formulation 5.

### 2.7 Sensory evaluation

Sensory evaluation followed ISO 8586 (2023) and ISO 6658 (2017). Fifty female students and staff of Sokoine University of Agriculture (SUA) were screened for sensory acuity using taste recognition, odor, and triangle discrimination tests (ISO 4120), yielding a final panel of 30 semi-trained assessors. Those retained were women aged over 20 years who were mothers or had practical experience caring for young children, free from allergies and respiratory infections, non-smokers, and who consented to participate. The retained assessors received two orientation sessions on the attributes and the rating scale. Each of the two developed formulations (F1 and F5) was prepared in three flavor variants and assigned a random three-digit sample code (SC) for blind evaluation, giving six coded samples: SC-777, SC-125 and SC-348 (Formulation 5) and SC-637, SC-861 and SC-999 (Formulation 1); SC denotes the blind sensory sample code and does not represent a distinct formulation (Figure 2). Porridges were reconstituted by dissolving 500 g of flour in 2000 mL of hot water, stirred to a uniform consistency, and served at room temperature in the coded cups. In each booth, all six coded cups were presented, with the presentation order randomized and balanced. Color, taste, aroma, texture, and overall acceptability were rated using a nine-point hedonic scale (1 = dislike extremely, 9 = like extremely), and mean scores were calculated for statistical analysis.

**Figure 2:**
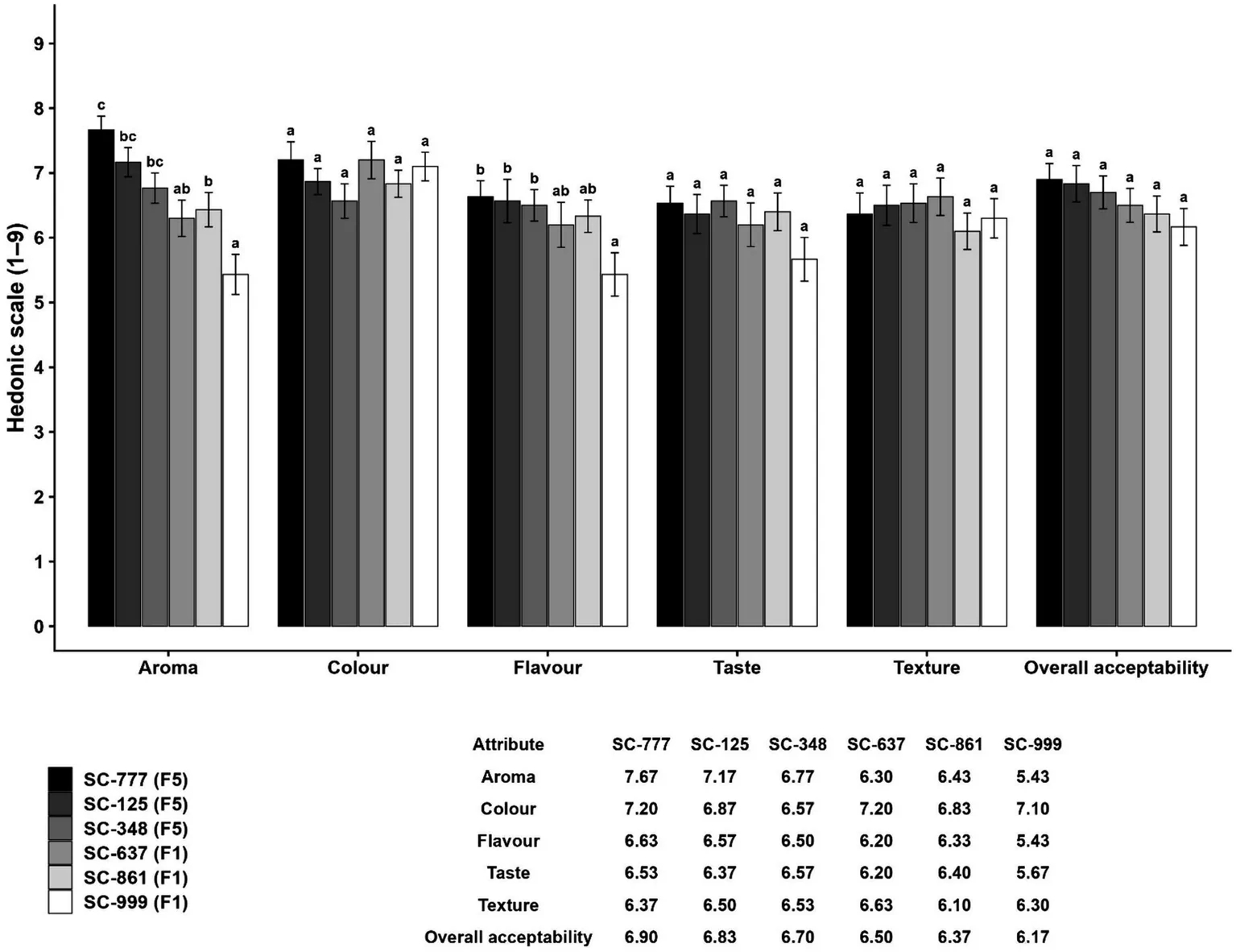
Sensory evaluation results presentation from six coded samples of two formulations (1&5).

### 2.8 Statistical analysis

All experimental data were analyzed using Gen-Stat software (Release 16.1, VSN International Ltd., UK) and graphical visualization were performed in R version 4.5.2 (2025). Data quality control was conducted throughout the study, and corrections were applied where necessary to ensure accuracy and consistency. For instance, during the water absorption capacity (WAC) and Bulk Density measurements, sample weights/volume were carefully corrected in triplicate for both extruded and un-extruded samples. Analysis of variance (ANOVA) was used to evaluate the effects of treatments according to the respective experimental designs. For WAC, a two-way ANOVA was performed with sample type and formulation as treatment factors. Sensory evaluation data were analyzed using a randomized complete block design, with panelists treated as blocks and product formulations as treatments. Where significant differences were detected, means were separated using Tukey’s Honest Significant Difference (HSD) test at a 5% probability level (*p* < 0.05). Sensory scores were also summarized descriptively to complement the statistical analysis.

## 3 Results

### 3.1 Water absorption capacity

The WAC varied significantly (*p* < 0.001) among sample types and formulations (Figure 3). Extruded samples exhibited significantly higher WAC values than un-extruded samples. Extruded formulation 5 (ExF5) recorded the highest mean WAC (2.64 ± 0.03 g/g), followed by extruded formulation 1 (ExF1) (2.38 ± 0.03 g/g). Un-extruded formulation 5 (UnF5) and un-extruded formulation 1 (UnF1) showed lower WAC values of 1.74 ± 0.03 g/g and 1.60 ± 0.02 g/g, respectively. This finding indicate that extruded samples absorbed more water and therefore retained less unabsorbed (free water) after hydration than the un-extruded samples.

**Figure 3:**
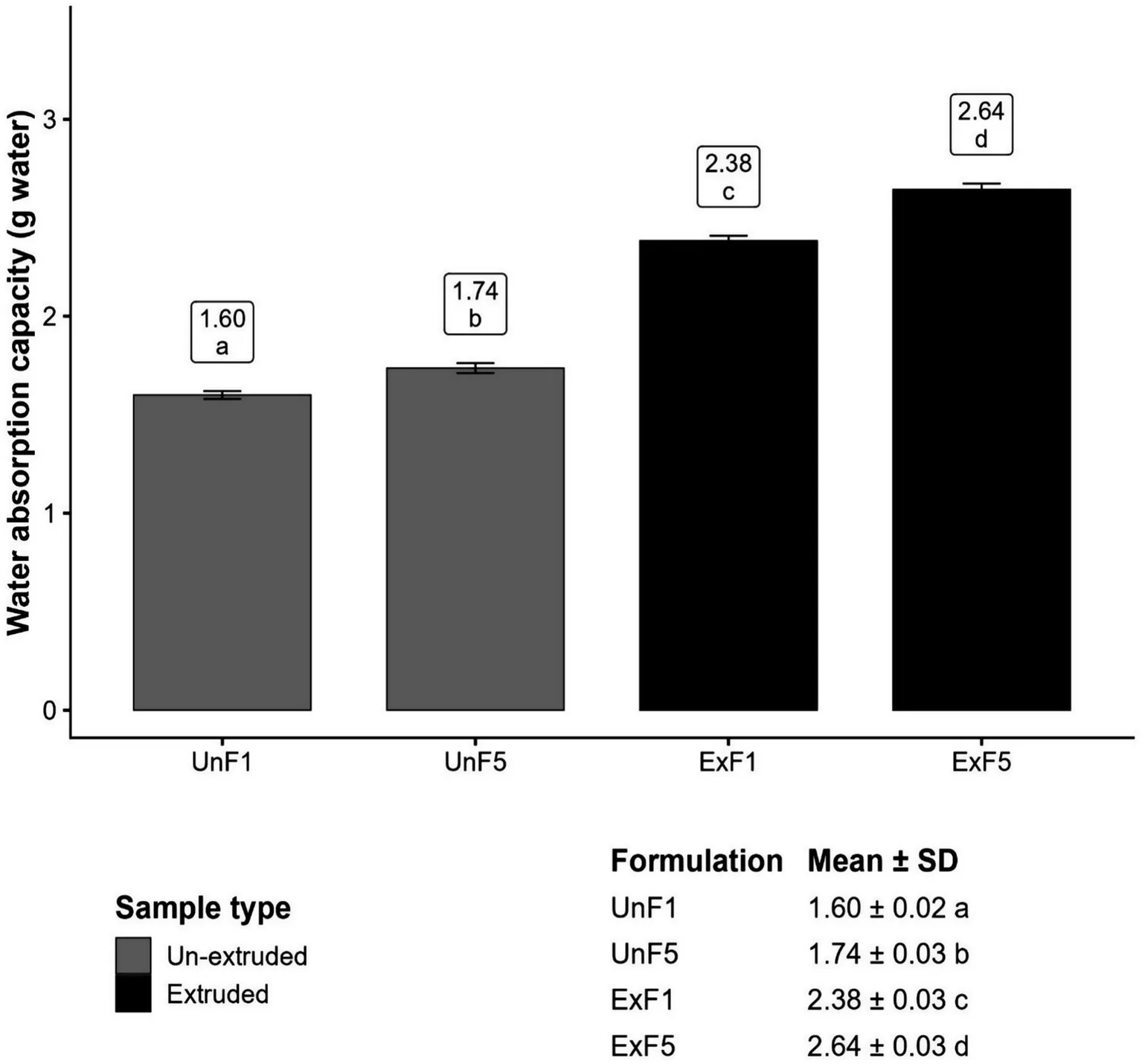
Water absorption capacity (g water/g flour) of un-extruded and extruded grain amaranth product samples.

### 3.2 Bulk density and flow behavior

Extrusion cooking significantly reduced the density of the composite flour (Table 3). Loose bulk density fell from 0.64 ± 0.02 g/mL in the uncooked flour to 0.38 ± 0.02 g/mL in the extruded flour, a reduction of 40.5% (*p* < 0.001), and tapped density fell correspondingly from 0.78 ± 0.04 to 0.43 ± 0.02 g/mL, a reduction of 45% (*p* < 0.001). The volume attained after tapping was higher in the extruded flour (88.3 ± 2.9 mL) than in the uncooked flour (81.7 ± 2.9 mL), indicating less possibility for further packing down any tighter. Carr’s index decreased from 18.3 ± 2.9% to 11.7 ± 2.9% (*p* = 0.047) and the Hausner ratio from 1.23 ± 0.04 to 1.13 ± 0.04 (*p* = 0.048), moving the material from the fair flowing into the good flowing category (27).

**Table 3:** Functional properties of the uncooked composite flour and the extruded product (27).

Parameter	Uncooked composite flour	Extruded product	*p*
Measured quantities			
Mass of empty cylinder, W₁ (g)	458.81	458.81	
Mass of cylinder + sample, W₂ (g)	522.37 ± 1.55	496.64 ± 1.63	
Mass of sample (g)	63.56 ± 1.55	37.83 ± 1.63	<0.001
Loose volume, V (ml)	100	100	
Tapped volume, V_t_ (ml)	81.7 ± 2.9	88.3 ± 2.9	0.047
Functional indices			
Bulk density (g/ml)	0.64 ± 0.02	0.38 ± 0.02	<0.001
Tapped density (g/ml)	0.78 ± 0.04	0.43 ± 0.02	<0.001
Carr’s index (%)	18.3 ± 2.9	11.7 ± 2.9	0.047
Hausner ratio	1.23 ± 0.04	1.13 ± 0.04	0.048

### 3.3 Proximate and mineral contents analysis

The proximate composition of the raw materials presented in Table 4. Grain amaranth contained high protein (17.46%), moderate fat (6.05%), and 11.17% moisture per 100 g, confirming its suitability as a base ingredient for energy-dense formulations for malnourished children. Soybean and sesame contributed the highest protein (38.29 g/100 g) and fat (50.48 g/100 g) fractions respectively, while maize provided the principal carbohydrate fraction at the lowest protein and fat contents of the four materials. The composition of the individual ingredients therefore determines the extent to which each formulation could reach the nutrient density required of a supplementary food.

**Table 4:** Proximate content for raw materials.

Material	Protein	Fat	Fiber	Ash	Moisture
Amaranth	17.46 ± 0.41*	6.05 ± 0.30	4.18 ± 0.05	2.88 ± 1.61	11.17 ± 0.26
Maize	11.13 ± 0.01	3.33 ± 0.08	2.17 ± 0.01	1.14 ± 1.24	9.33 ± 1.23
Soy bean	38.29 ± 0.37	17.41 ± 0.15	6.01 ± 0.02	5.18 ± 0.83	7.90 ± 1.27
Sesame	25.46 ± 0.70	50.48 ± 0.56	8.77 ± 0.01	6.28 ± 2.46	5.27 ± 2.18

*Mean ± SD.

The analyzed composition of the formulated products is presented in Table 2. Analyzed protein exceeded the theoretical estimates of the formulation matrix (Table 1), which projected 14.6 g/100 g for Formulation 1 and 16.2 g/100 g for formulation 5, a difference attributable by moisture loss during extrusion and the consequent concentration of nutrients on a dry matter basis. Formulation 5 recorded higher values than Formulation 1 for energy (391.93 against 379.67 kcal/100 g), protein (20.53 against 18.14 g/100 g) and fat (11.95 against 8.75 g/100 g). Formulation 1 attained 94.9% of the specified minimum energy density and 97.2% of the minimum fat content, falling below specifications on both counts, whereas Formulation 5 satisfied the fat requirement and nearly approached the energy requirement (98%). Formulation 5 was therefore selected for full compositional characterization, for compliance against the WFP/UNICEF Super Cereal Plus (CSB++) specification (Table 5), and for the sensory evaluation study reported subsequently.

**Table 5:** Proximate and mineral composition of ENDIP formulation 5 relative to the WFP/UNICEF Super Cereal Plus (CSB++) specification (36, 37).

Component (unit)	ENDIP F5	CSB++ specification	Assessment
Proximate composition			
Energy (kcal/100 g)	391.93	≥ 400	Marginally below (98.0%)
Protein (g/100 g)	20.53	≥ 16.0	Exceeds
Fat (g/100 g)	11.95	≥ 9.0	Exceeds
Mineral composition			
Calcium (mg/100 g)	587.86	420–660	Within range
Iron (mg/100 g)	11.44	9.0–14.8	Within range
Zinc (mg/100 g)	8.98	8.0–14.0	Within range
Sodium (mg/100 g)	226.57	≤ 270 (ceiling)	Compliant (83.9% of ceiling)
Potassium (mg/100 g)	240.92	650–1,050	Below specification (37.1%)

CSB++ = Super Cereal Plus, a fortified corn–soy–milk blend.

Formulation 5 was richer in iron and potassium, Formulation 1 in calcium and magnesium, with zinc comparable between the two (Table 2), differences traceable to ingredient ratios rather than processing. Raising one nutrient-dense ingredient therefore improved some minerals at the expense of others. Formulation 5 still met the CSB++ targets for calcium, iron and zinc without any added mineral mix, and its sodium stayed below the limit. Only potassium fell short, because none of the four ingredients is a good source of it.

### 3.4 Anti-nutrient load

The concentrations of tannins, oxalates and phytate in the raw materials and blended formulations are presented in Table 6. Among the raw ingredients grain amaranth was highest in phytate, sesame in oxalates and soybean in tannins, while pro- vitamin A maize was lowest across all three analytes. Blending reduced the anti-nutrient load of the F5 composite by 28.1% for tannins, 48.6% for oxalates, and 56.1% for phytate relative to raw grain amaranth. Extrusion lowered them further by 30.7, 31.7, and 48.9% for tannins, oxalates and phytate respectively, giving cumulative reductions of 50.2, 64.9, and 77.6% (Figure 4). F1 followed the same pattern, with phytate declining by 67.5%, confirming phytate as the analyte most sensitive to processing.

**Table 6:** Anti-nutrients load.

Sample	Tannin	Oxalate	Phytate	Units
Pro-vitamin maize	4.83 ± 0.11*	21.72 ± 0.12	11.49 ± 0.04	mg/100 g
Amaranth	51.22 ± 0.11	1263.44 ± 10.82	1830.80 ± 3.56	mg/100 g
Soybean	72.11 ± 0.15	339.26 ± 3.94	219.07 ± 10.09	mg/100 g
Sesame	4.53 ± 0.11	1368.87 ± 6.55	457.28 ± 1.42	mg/100 g
Blended formulations				
F1- raw	32.14 ± 0.21	571.37 ± 0.32	701.68 ± 2.04	mg/100 g
F5- raw	36.83 ± 0.14	649.27 ± 0.14	803.02 ± 1.06	mg/100 g
F1-extruded	31.08 ± 0.04	475.13 ± 6.69	228.12 ± 1.91	mg/100 g
F5-extruded	25.513 ± 0.61	443.419 ± 3.43	410.286 ± 2.320	mg/100 g

*Mean ± SD.

**Figure 4:**
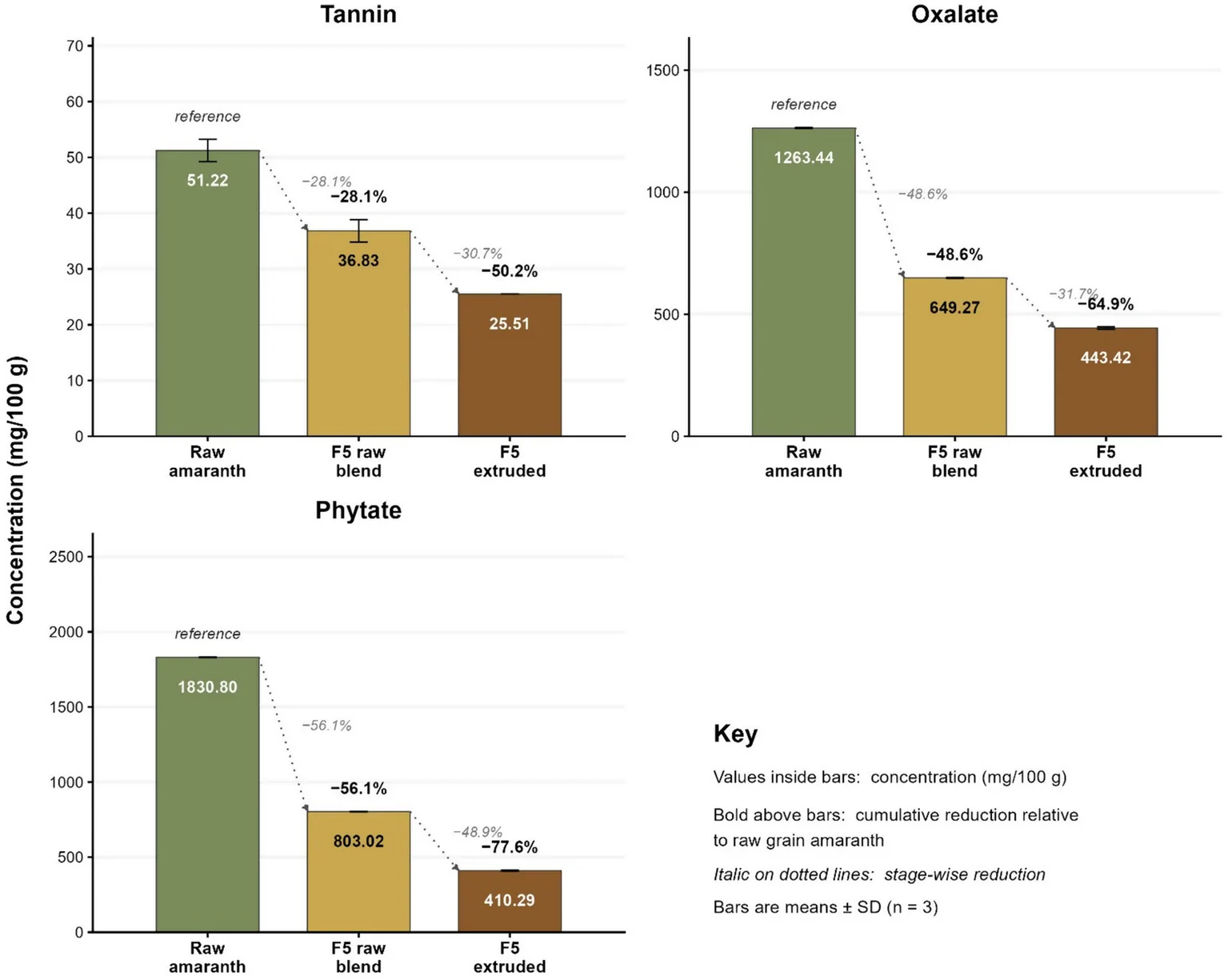
Stage wise reduction of tannin, oxalate and phytate from raw amaranth to extruded F5.

Phytate to mineral molar ratios calculated from the phytate concentration Table 6 and the mineral concentration in Table 2 are presented in Table 7. Extrusion lowered the phytate to zinc molar ratio of Formulation 5 from 8.86 in the raw blend to 4.53 in the extruded product, and the phytate to calcium ratio from 0.083 to 0.042. The phytate to iron ratio fell from 5.94 to 3.03 in Formulation 5 but remained above unity in both extruded products.

**Table 7:** Phytate to mineral molar ratios of the raw and extruded blends (42).

Molar ratio	Critical value	F1 raw	F1 extruded	F5 raw	F5 extruded	Assessment of extruded products
Phytate: Fe	<1	10.93	3.55	5.94	3.03	Not met; iron absorption likely inhibited
Phytate: Zn	<5	7.75	2.52	8.86	4.53	Met; zinc absorption adequate
Phytate: Ca	<0.24	0.067	0.022	0.083	0.042	Met; calcium absorption not compromised

### 3.5 Sensory evaluation

Product acceptability (Figure 2) showed that formulation had a significant effect on aroma (*p* < 0.001) and flavor (*p* = 0.002). In contrast, color, taste, texture, and overall acceptability were not significantly different (*p* > 0.05). Aroma scores ranged from 5.43 to 7.67, with SC-777 scoring highest and SC-999 lowest. Flavor scores varied from 5.43 to 6.63, with SC-777, SC-125, and SC-348 performing better than SC-999. Differences in color, taste, and texture were minimal across all samples. Overall acceptability ranged from 6.17 to 6.90, with SC-777 rated highest and SC-999 lowest. Figure 2 summarizes these sensory patterns across formulations.

## 4 Discussion

This study contributes to efforts aimed at developing nutritious, locally sourced complementary foods for the prevention and management of MAM among children in Tanzania. Initially, five formulations were developed; however, based on linear programming optimization technique, Formulations 1 and 5 were selected for further processing and chemical analysis. This selection reduced processing complexity and extrusion-related costs while retaining formulations with acceptable sensory properties. Therefore, subsequent analyses focused on the functional and nutritional characteristics of these two formulations, particularly WAC, Bulk density and nutrient composition. The findings demonstrate that both ingredient composition and processing technique significantly influenced the functional quality and nutrients bioavailability of the developed products.

### 4.1 Theoretical formulation and the selection of formulation 1 and 5

Linear programming was used instead of trial and error blending approach, so that the objective, constraints and feasible region were fixed in advance, making the formulation decision explicit and reproducible. Linear programming is well established in complementary and therapeutic food formulation (28); in amaranth specifically, Akande et al. (29); used least-cost software to generate six instant-porridge formulations before hedonic screening. Here, cost served as a screening criterion rather than the optimization objective, so least-cost solutions could not displace the nutrient-dense ingredients central to the product. The procedure generated five feasible formulations covering a narrow compositional range: energy from 369.3 to 384.9 kcal/100 g, protein from 14.6 to 16.6 g/100 g, fat from 9.6 to 12.8 g/100 g, grain amaranth inclusion from 31 to 41%, and raw-material cost from 192.8 to 215.9 TZS/100 g (Table 1). Because all five satisfied the nutritional constraints, the choice among them was determined by their position within the feasible region rather than by nutritional adequacy alone. Formulation 1 was retained as the least-cost solution (192.8 TZS/100 g) meeting every constraint, representing the most economically realistic candidate for community-level production.

Formulation 5 was retained on two grounds. First, it was the most nutrient-dense solution within the technologically feasible set, recording the highest crude protein (16.2 g/100 g) and the highest energy density of any formulation satisfying the fat ceiling, at a grain amaranth inclusion of 40%. Second, although Formulations 2 and 4 carried marginally higher grain amaranth inclusion than Formulation 5 (41% against 40%), both were less nutrient-dense, recording crude protein of 14.9 and 15.3 against 16.2 g/100 g and lower energy density (370 kcal/100 g for both, against 372.1 kcal/100 g for Formulation 5) at raw-material costs of 205.4 and 207.7 TZS/100 g. The additional 7.1 to 9.4 TZS/100 g required to move from Formulation 4 or 2 to Formulation 5 therefore purchased 0.9 to 1.3 g/100 g of further protein together with a higher energy density, a trade the intended application justifies: the volume of gruel a child with MAM can consume, rather than the cost of the raw materials, is the binding constraint on nutrient delivery (30). Retaining Formulations 1 and 5 together bracketed the feasible region at its cost and nutrient-density boundaries, allowing the trade-off between them to be evaluated experimentally rather than assumed.

Formulation 3 recorded the highest theoretical protein (16.6 g/100 g) and energy (384.9 kcal/100 g) but was excluded on technological grounds. Its fat content of 12.8 g/100 g, arising from a sesame inclusion of 11%, exceeded the fat ceiling of 11.0 g/100 g imposed on the model to limit barrel slippage and the associated loss of specific mechanical energy during twin-screw extrusion, and to contain the risk of oxidative rancidity in a product intended for unrefrigerated distribution (29, 31); the marginal protein gain of 0.4 g/100 g over Formulation 5 did not justify that exposure. Formulation 3 also carried the highest raw-material cost (215.9 TZS/100 g) and the lowest grain amaranth inclusion (31%), making it simultaneously the most expensive formulation and the least aligned with the amaranth-utilization objective.

### 4.2 Functional properties: water absorption capacity and bulk density

Water absorption capacity (WAC) and bulk density in an instant porridge they describe two aspects of the same structural change and are best interpreted together. Extrusion increased water absorption capacity in both formulations (*p* < 0.001) while reducing loose bulk density by 40.5% and tapped density by 45%. Both align with a single mechanism; the temperature, pressure and shear forces in the extruder barrel disrupt the semi-crystalline organization of starch granules and unfold the protein matrix, and flash evaporation at the die melt into a porous, low density structure with greatly increased surface area of gelatinized starch available for hydrogen bonding (32).

The bulk density of the extruded product (0.38 ± 0.02 g/mL) is definitely lower than 0.59 g/mL reported by Atukuri et al. (31), for optimized instant grain amaranth flour. Because a lower bulk density permits a higher nutrient load per unit volume of reconstituted gruel, this difference is nutritionally favorable rather than incidental, and is the property that most directly addresses the limitation of traditional cereal gruels, which forms viscous pastes requiring dilution that reduces their nutrient density (33).

Formulation modulated the magnitude of the effect. Formulation 5 recorded higher water absorption capacity than Formulation 1 in both the uncooked (1.74 ± 0.03 versus 1.60 ± 0.02 g/g) and extruded states (2.64 ± 0.03 versus 2.38 ± 0.03 g/g), consistent with its higher amaranth and lower maize inclusion; amaranth protein carries a greater proportion of polar residues than maize zeins (protein) (12), and protein-rich matrices bind water more strongly through these groups (34). The flow indices carry a distinct meaning. Carr’s index fell from 18.3 ± 2.9% to 11.7 ± 2.9% (*p* = 0.047) and the Hausner ratio from 1.23 ± 0.04 to 1.13 ± 0.04 (*p* = 0.048), relocating the material from the fair-flowing to the good-flowing category (27). That extrusion improved flow while also improving reconstitution is notable, since the reduced bulk density accompanying expansion would ordinarily be expected to increase inter-particle cohesion (35).

### 4.3 Nutritional adequacy: proximate and mineral composition

Proximate and mineral composition are treated jointly because the same formulation decisions drive both and because compliance with a supplementary-food specification is assessed across the two simultaneously. Analyzed composition exceeded the values projected by the linear-programming matrix, attributable to moisture loss during extrusion and the consequent concentration of nutrients on a dry-matter basis. Formulation 5 was nutritionally superior to Formulation 1 across all three macronutrient measures, recording higher energy (391.93 versus 379.67 kcal/100 g), protein (20.53 versus 18.14 g/100 g) and fat (11.95 versus 8.75 g/100 g).

Compliance was assessed against the WFP/UNICEF Super Cereal Plus (CSB++) specification, which is the appropriate comparator for a fortified blended food intended for MAM (36, 37). Formulation 5 exceeded the protein, fat and achieved 98.0% of the energy minimum. The protein surplus is meaningful rather than nominal: plant proteins are digested less completely than animal-source proteins, and a margin above specification offsets the reduction in utilizable protein imposed by lower digestibility and residual anti-nutritional factors (38).

Mineral composition of Formulation 5 reflected this pattern. Calcium (587.86 mg/100 g), iron (11.44 mg/100 g) and zinc (8.98 mg/100 g) all fell within the CSB++ ranges without external mineral fortification, indicating that a carefully constructed whole-food blend can reach micronutrient targets normally met by premix addition (39). Sodium remained at 83.9% of the permitted ceiling, favorable given the renal solute constraints of young children. Potassium was the single exception, at 240.92 mg/100 g or 37.1% of the lower specification bound. This is a genuine limitation rather than an analytical artefact: none of the four ingredients is potassium-dense, and the deficit cannot be closed by adjusting their ratios within the constraints imposed by extrusion. Potassium adequacy matters particularly in MAM because intracellular potassium is depleted during wasting and its repletion is required for restoration of cell membrane function and lean tissue accretion (40). Because the CSB++ formulation itself meets its potassium specification through deliberate addition of potassium chloride (36), the most direct remedy is targeted fortification of the blend with a permitted potassium salt at a level calculated to reach the specification bound, a well-established and low-cost adjustment that does not require reformulation of the ingredient base.

For practical application, this composition is intended to be delivered as a 100 g/day ration. At the energy density of Formulation 5 (391.93 kcal/100 g), 100 g supplies approximately 392 kcal/day; reconstituted with hot water at about a 1:4 (w/v) flour-to-water ratio, it yields roughly 500 mL of porridge, fed in two to three portions across the day to complement breastmilk and the home diet. This ration provides a meaningful energy and micronutrient supplement while remaining within the volume a child with MAM can realistically consume, and it is the basis on which the compositional adequacy discussed above translates into daily nutrient delivery (16, 17).

### 4.4 The analysis of anti-nutrient load

The mineral concentrations reported here reflect what the product contains rather than what a child actually absorbs, and in plant-based foods that difference matters, because phytates, tannins and oxalates bind divalent cations and hold them out of reach (26). The difficulty is built into the formulation; grain amaranth supplies most of the iron and magnesium, but it also supplies most of the phytate (14), so the very choice that enriched the product also worked against its uptake. We addressed this in two stages. Blending first diluted the amaranth load, cutting tannins by 28.1%, oxalates by 48.6% and phytates by 56.1% relative to raw grain amaranth figures in formulations. Extrusion then removed a further 30.7, 31.7 and 48.9% (Figure 4).

These extrusion figures fall within the published range; cereal bran, for instance, has shown drops of 54.5% in phytic acid and 36.8% in oxalates (41). Against our 48.9 and 31.7%. That the results are typical rather than exceptional is reassuring, since it suggests they should hold up under comparable conditions elsewhere. What stands out is the combined effect: acting in sequence on the same load, the two steps together achieved reductions of 50.2, 64.9, and 77.6% from the raw baseline, with the largest fall in phytate; the compound that binds iron and zinc most tightly.

Molar ratios (Table 7) show whether the minerals are actually free to be absorbed: the more phytate present relative to a mineral, the less of that mineral the body can take up. The signs were mostly encouraging. In Formulation 5 the phytate: zinc ratio fell from 8.86 to 4.53, below the critical value of 5 (26), and phytate: calcium (0.042) stayed far under the 0.24 threshold (42), so zinc and calcium uptake should be adequate. Iron was the exception. Phytate: iron held at 3.03 (F5) and 3.55 (F1), above 1 (26, 43), leaving iron the limiting mineral. The practical answer is fortification by adding ascorbic acid at 2:1 or higher to iron, or NaFeEDTA, would free iron for absorption (26, 44).

### 4.5 The product acceptability study

Compositional adequacy is necessary but not sufficient: a supplementary food that children will not consume delivers no nutrients. Formulation significantly affected aroma (*p* < 0.001) and flavor (*p* = 0.002), while color, taste, texture and overall acceptability did not differ significantly (*p* > 0.05). Overall acceptability ranged from 6.17 to 6.90 on the nine-point scale, placing all samples above the neutral point. This range corresponds closely with published values for comparable products: Atukuri et al. (31), reported a sensory acceptability score of 6.69 for optimized instant grain amaranth flour, effectively identical to the 6.90 recorded for extruded Formulation 5 here. ENDIP is therefore acceptable at a level typical of amaranth-based instant porridge.

Extruded Formulation 5 (SC-777) achieved the highest aroma score and ranked among the best performers for flavor and overall acceptability. Because the formulations advanced to extrusion were selected on linear-programming grounds alone, the sensory data provide an independent check on that selection rather than a component of it. The aroma and flavor advantage is plausibly attributable to the added coconut flavoring, which masks the beany and cereal off-notes commonly reported in composite flours (45), reinforced by the mild nutty character contributed by the higher amaranth inclusion (46). These observations agree with earlier reports that flavor enhancement and the masking of beany or cereal off-notes improve the acceptability of fortified complementary foods among caregivers and children alike (38). That color, taste and texture did not differ here indicates the two formulations were physically comparable, so the observed preference reflects volatile rather than structural differences.

### 4.6 Study limitations

Several limitations qualify these findings. The study characterized composition and acceptability but did not test clinical efficacy, so ENDIP’s capacity to reverse MAM is inferred from compliance with the CSB++ specification rather than demonstrated. Mineral bioavailability was estimated from phytate-to-mineral molar ratios rather than measured directly, and because the colorimetric assay quantifies total phytate without distinguishing inositol hexa-phosphate from its degradation products, the post-extrusion ratios likely overstate the inhibitory fraction present. Functional testing was limited to water absorption capacity and flow indices. Potassium remains below specification, and no fortification strategy has been validated. Shelf-life, microbiological stability and lipid oxidation are addressed in a separate companion paper. Finally, the sensory panel comprised adult female caregivers rather than children in the target age group.

## 5 Conclusion

This study demonstrated that locally available ingredients such as grain amaranth, pro-vitamin A maize, and legumes can be used to develop nutrient-dense instant complementary foods with potential application in the management of moderate acute malnutrition (MAM). Among the developed products, Formulation 5 showed the most promising balance of nutritional quality, functional properties, reduced anti-nutritional factors, and consumer acceptability. Extrusion processing further enhanced product quality by improving water absorption capacity and reducing anti-nutritional compounds, thereby potentially improving nutrient availability. The findings highlight the potential of food-to-food fortification using local crops as a sustainable and affordable alternative to imported supplementary foods while supporting local food systems and nutrition security. However, further studies are needed to evaluate clinical efficacy, micro-nutrient bio-availability, and cost-effectiveness before large-scale application and policy adoption.

## Data Availability

The raw analytical and sensory datasets underlying this study are openly available in the Zenodo repository under a (Creative Commons Attribution 4.0 International CC BY 4.0) license: https://doi.org/10.5281/zenodo.21678598. Further inquiries can be directed to the corresponding author.
